# Common Colds as a Possible Source of Contagion for Lobar Pneumonia

**Published:** 1918-11

**Authors:** 


**Common Colds as a Possible Source of Contagion for Lobar Pneumonia.** By EUGENIA VALENTINE, *Journal of Experimental Medicine.* Jan. 1, 1918.

A series of common colds was examined to determine the types of pneumococci present. This was undertaken not so much from the standpoint of determining the etiological significance of pneumococci in common colds, but rather to determine whether the fixed types occurred. If these were found, common colds would then have to be considered as a source of infection in the spread of lobar pneumonia.

By injecting the nasal secretion or sputum into mice, pneumococci were recovered in 37 out of 65 cases of common colds. By direct plating on blood agar, pneumococci were found in 6 additional cases. Of the total 43 cases, the incidence of type or group of pneumococcus recovered was as follows : Group I, 2 cases; Group II, 2 cases; Group III, 4 cases; Group IV, 35 cases. The cases from which Group II was isolated, and one of the Type III cases, had been in contact with pneumonia, and it is impossible to decide whether they were merely contact carriers, or whether the cold was an actual infection by pneumococci due to the contact with pneumonia cases.

In determining the type, immune horse sera were employed. Considerable agglutination was encountered in the case of either or both of the horse sera (Type I and Type II) in low dilutions with the strains finally included in Group IV.

In three of the four cases yielding Type III, mouse inoculation only was done. In the fourth, direct cultures showed the presence of about 75 o/o of this organism. Pneumococcus mucosus has been found by other observers in the secretion of the respiratory tract in conditions other than lobar pneumonia, as well as in otherwise normal individuals. All these individuals, therefore, must be looked upon as potential sources of infection for others, or as sources of possible autogenic cases of lobar pneumonia.

The recovery of pneumococci of Type I from two cases of common colds raises the question whether these persons were carriers, or whether the inflammation was due to the pneumococcus.

In these two instances, with no known contact with cases of pneumonia, pneumococcus Type I was found to be the predominating organism, which strongly suggests that it was the etiological agent in these colds. If this is so, common colds of this type must be looked upon as a possible source of contagion in the development of lobar pneumonia, due to the Type I pneumococcus.

